# Stakeholders’ Perspectives on eHealth Support in Colorectal Cancer Survivorship: Qualitative Interview Study

**DOI:** 10.2196/28279

**Published:** 2021-09-07

**Authors:** Anne Marie Lunde Husebø

**Affiliations:** 1 Research Group of Nursing and Health Sciences Stavanger University Hospital Stavanger Norway; 2 Department of Public Health Faculty of Health Sciences University of Stavanger Stavanger Norway

**Keywords:** cancer patients, carers, colorectal cancer, digital competence, eHealth, health care professionals, follow-up service, web-based information seeking, self-management support, treatment burden, mobile phone

## Abstract

**Background:**

eHealth interventions may represent the way forward in following up patients with colorectal cancer (CRC) after hospital discharge to support them in coping with the illness, strengthen their self-management, and increase their quality of life. By involving end users of eHealth in cocreation processes when designing eHealth solutions, an acceptable and relevant product can be secured. Stakeholders’ perspectives could aid in closing the gap between research-developed products and the implementation of eHealth services in real-life scenarios.

**Objective:**

This study aims to explore the views of patients with CRC, their informal caregivers, and health care professionals (HCPs) on information technology and the design of eHealth support in CRC care.

**Methods:**

A qualitative, explorative design was used to conduct 31 semistructured individual interviews with 41% (13/31) patients with CRC, 29% (9/31) informal caregivers, and 29% (9/31) HCPs recruited from the gastrosurgical ward of a university hospital in southwestern Norway. A semistructured interview guide was used for data collection, and the data were analyzed by systematic text condensation.

**Results:**

Participants described the diverse experiences of patients with CRC seeking web-based information. Age and digital competence were highlighted as influencers of the use of information technology. Patients rarely received advice from HCPs about relevant and secure websites containing information on CRC diagnosis and treatment. Features of desired eHealth interventions in following up patients with CRC were patient education, health monitoring, and communication with HCPs.

**Conclusions:**

Several elements affect the activities of patients with CRC seeking health information. Age, inexperience with computer technology, and lack of access to web-based health information may reduce the ability of patients with CRC to engage in decision-making processes regarding illness and treatment. An eHealth service for patients with CRC should comprise features for information, education, and support for self-management and should aim to be individually adapted to the patient’s age and digital competence. Involving end users of eHealth services is necessary to ensure high-quality tailored services that are perceived as user friendly and relevant to the end users.

## Introduction

The World Health Organization [1] defines eHealth as “the use of information and communication technologies (ICT) for health.” The development of eHealth solutions in health care services is a growing field of interest in academic and clinical research. In cancer care, eHealth interventions are designed to help patients cope with cancer and treatment side effects, strengthen self-management, and improve their quality of life (QoL) [2]. QoL consists of physical, psychological, social, spiritual, and environmental values [3]. Cella and Tulsky [4] proposed a definition of QoL for use in cancer care that includes the patient’s own preferences into the level of impairment: “patients’ appraisal of and satisfaction with their current level of functioning compared with what they perceive to be possible or ideal [p. 329].”

Colorectal cancer (CRC) is among the most frequent cancer diagnoses worldwide, with nearly 2.0 million new cases in 2018 [5]. Follow-up of curatively treated patients with CRC involves recurrence surveillance and prevention, health maintenance, and psychosocial support [6]. A decrease in postoperative length of stay during primary surgical treatment has been observed for patients with CRC [7]. Many patients may experience feelings of emptiness and insecurity without professional care when they return home after hospitalization [8]. In the follow-up phase, eHealth tools, such as mobile apps, can be used for self-reporting of side effects of adjuvant CRC treatments [9], to enhance the capacity to self-manage and increase QoL [10] and help patients to access social media (eg, Facebook, Twitter, cancer survivor networks) for information and peer support [11]. For patients to be able to access eHealth tools, they need digital competence, including information and data literacy (ie, ability to search, filter, evaluate, and manage digital content), and communication and collaboration skills (ie, interaction, sharing, netiquette, and digital identity) [12].

Patients with CRC have reported an extensive and prolonged need for information and knowledge about their cancer diagnosis, treatments, and prospects [13]. The internet is an increasingly important source of health-related information for gaining increased knowledge and the ability to engage in health care decisions [14]. In Norway, 96% of the households have internet access; 95% of the population in all regions uses the internet daily; and, on average, there are nearly 8 devices with internet access per household [15]. A recent study on eHealth use among 18,500 Norwegians aged more than 40 years showed that nearly 53% of the participants had used an eHealth service during the last year and that eHealth use was positively influenced by younger age, being a woman, or having higher education or higher socioeconomic status [16]. Cancer patients have been found to use the internet for health information from the time of their diagnosis, and they continue to use it through their survivorship [17]. Issues for which cancer patients use the internet to gain information include cancer diagnosis, cancer treatment and side effects, health insurance and financial issues, and genetics and heritability [18]. Although many cancer patients consult web-based sources for health information, research shows that they use their oncologists or other relevant health care professionals (HCPs) as their primary source of information. This gives the HCP an opportunity to provide their patients with information on reliable websites [19].

The growing number of patients with CRC requires a more flexible and dynamic follow-up approach for curative CRC treatment [20]. To help meet support needs of patients with CRC during the vulnerable period in which they manage a changed life situation following a cancer diagnosis, eHealth interventions using smart applications may be one way forward [1]. Health information is an important feature of eHealth, and eHealth services are perceived as safe and reliable sources of health information [21]. eHealth is closely connected to social innovations, where digital solutions are developed in cocreation processes with end users such as patients and HCPs with the aim of creating new, improved, and efficient health care services [22]. The implementation of eHealth interventions is challenged by a gap between research-produced innovations and the actual use of such innovations in clinical practice [23]. To close the gap, cocreation processes are suggested in the design of eHealth applications to ensure that the end product will meet the needs of technology users and contribute to desired outcomes [24]. The aims of this study are to explore how patients with CRC, their informal caregivers, and HCPs with experience in CRC treatment and care relate to web-based health information and map out their thoughts on future eHealth services to improve self-management in CRC care.

The research questions developed were as follows:

1. What is the experience of patients, informal caregivers, and HCPs with information and communication technology (ICT) for CRC management?
2. How should eHealth services be designed, and which needs should they meet in supporting patients with CRC following primary surgical treatment?

## Methods

### Design

This was a qualitative, explorative study that used semistructured individual interviews [25] to explore experiences of ICT among patients with CRC, their informal caregivers, and HCPs. Data were collected on the use behaviors of ICT (eg, internet, smart apps, electronic medical journals) from the time of diagnosis, during surgical treatment and after hospital discharge, and on preferences and desired outcomes for future eHealth applications.

Ethical approval was provided by the National Committee for Research Ethics in the Social Sciences and Humanities (No. 2017/284) and by the university hospital research ethics board. All participants provided informed consent for participation in the study.

### Eligibility and Recruitment

Eligible participants were adult patients (aged 18-80 years) diagnosed with CRC and surgically treated with curative intent and their adult informal caregivers, as appointed by the patient. In addition, the study recruited HCPs with more than 1 year of experience in CRC treatment and care in a surgical ward. All participants were required to understand and speak Norwegian. Patients and their informal caregivers were recruited by a study nurse at a surgical outpatient clinic at a university hospital in Norway who provided them with oral and written information on the study purpose. A staff nurse at a gastroenterological surgical ward at the aforementioned university hospital recruited the HCPs. All the patients who were approached agreed to participate. Reasons for nonparticipation among informal caregivers and HCPs were not recorded. One HCP withdrew consent because of a changed work schedule.

A model of information power by Malterud et al [26] guided the total number of interviews, indicating a narrow study aim: participants experienced with CRC treatment and care, a strong dialog during interviews, clear theoretical underpinnings, and an appropriate thematic analysis.

### Data Collection

Data collection was carried out as part of a larger interview study on the transition from hospital to home and the follow-up needs of patients with CRC. Single interviews with patients were carried out at the location of their preference, either in office facilities at the university hospital, at the university, or in the homes of patients. One of the interviews with informal caregivers was carried out at the university hospital, another in an informal caregiver’s home, and the rest by telephone. Interviews with HCPs were conducted at suitable locations in the university hospital. The interviews with patients, HCPs, and 2 of the informal caregivers were conducted by the author (AMLH), who is a nurse and associate professor (PhD) experienced in qualitative research. A professor of nursing experienced in qualitative research performed the majority of the interviews with informal caregivers. Both interviewers have research experience in the field of chronic and long-term illness and eHealth. A semistructured interview guide was used to guide the interviews, and the themes on eHealth and digital competence were informed by earlier research on digital information technology in a cancer survivorship context [13,17-20] (Textbox 1). The interviewer used follow-up questions, such as “Have I understood you correctly when you say...?” to confirm the interviewees’ answers. The interview guide was pilot-tested by a patient together with an informal caregiver and an HCP.

**Interview guide.**

**Colorectal cancer patients and informal caregivers—use of social media and information technology**

- What experience do you have of the use of technology such as mobile phones, tablets, PCs?
- Have you accessed websites or eHealth applications during the time of diagnosis or before or after surgical treatment, for example, an app on your mobile or social media?
- What type of health information do you envisage obtaining through internet sources or applications?
- What should such an eHealth service look like, and how should it function to support your self-management and information needs? Who would you like to be able to communicate with via an eHealth service (eg, other patients, relatives, your general practitioner, hospital personnel, support groups)?
- If you use information and communication technology, how do you use these to support your relative or spouse in self-management of illness and follow-up of treatment? (informal caregivers only)

**Health care professionals—technological information support**

- What is your impression of where patients obtain information related to illness and treatment?
- What is your impression of patients’ use of social media as support in disease management and follow-up of treatment?
- What benefit do you think patients gain from using social media?
- What type of health information do you envisage the patient receiving through an eHealth solution?
- What should such a technological aid look like, how should it function, and what features should be included?

Data collection continued until no new data emerged within each of the 3 study populations.

The interviews lasted for a total 35 to 90 minutes. The telephone interviews lasted shorter than face-to-face interviews. All interviews were audiotaped and transcribed verbatim by a health care secretary experienced in transcription for research purposes. To preserve anonymity, any information that might reveal a participant’s identity was removed during transcription. A total of 23,913 words were transcribed from interviews on eHealth and digital competence. The transcripts were uploaded to NVivo software (QSR International) [27].

### Data Analysis

A stepwise systematic text condensation guided the data analysis [28]. First, the transcripts were read repeatedly and comprehensively to gain an overall first impression and identify the preliminary themes. Second, deductive coding of meaning units (participants’ quotes) within each of the preliminary themes was performed. The deductive coding was based on an earlier work on eHealth concept development [1] and research on eHealth within cancer populations [13,17-20]. Third, the codes were sorted into categories, which formed the final main themes in the fourth step. The transcripts from each interview were arranged in 3 clusters (ie, HCPs, patients, and informal caregivers), and the clusters were then merged during coding in NVivo.

To achieve trustworthy results, the same researcher involved in the informal caregiver interviews validated the data analysis by reading a sample of the transcripts and coded data material. In an analysis meeting between the author and researcher, the categorization into final themes continued until agreement was reached.

Stepwise data analysis is shown in Multimedia Appendix 1. Findings constituting the 3 main themes derived from the data, *Seeking health information*, *Factors affecting the use of information technology*, and *Future eHealth services for colorectal patients*, with corresponding subthemes, are presented later. Participants’ quotes are provided to add documentary and aesthetic value to the findings [29].

## Results

### Participants

The study comprised a total of 31 participants: 41% (13/31) patients surgically treated for CRC, 29% (9/31) informal caregivers, and 29% (9/31) HCPs. The median ages were 65 years for patients (range 46-79 years), 68 years for informal caregivers (range 43-77 years), and 33 years for HCPs (range 22-52 years). The majority of the participants were women, with 4 being patients, 6 informal caregivers, and 7 HCPs. A total of 8 patients were diagnosed with colon cancer, whereas 5 were diagnosed with rectal cancer. Among the informal caregivers, 8 were spouses and 1 was an adult offspring. All but one informal caregiver lived with the patient. Information on reimbursement paid informal caregivers was not collected. The majority of HCPs were nurses (n=7). A total of 5 HCPs had 1 to 3 years of work experience in CRC treatment and care, whereas 4 had over 3 years of experience. Participants’ characteristics are presented in Table 1.

**Table 1:** Characteristics of the study sample (N=31).

Characteristic			Participants						
			CRC^a^ patients (n=13)		Informal caregivers (n=9)			HCP^b^ (n=9)	
Age (years), range			46-79		43-77			22-52	
**Sex, n (%)**									
	Male	9 (69)		3 (33)			2 (22)		
	Female	4 (30)		6 (66)			7 (77)		
**Diagnosis, n (%)**						N/A^c^			N/A
	Colon cancer	8 (61)							
	Rectal cancer	5 (38)							
**Educational status of patients and informal caregivers, n (%)**									N/A
	Primary school	3 (23)		1 (11)					
	High school	4 (30)		5 (55)					
	College or university	6 (46)		2 (22)					
	Missing	N/A		1 (11)					
**Informal** **caregiver** **relation, n (%)**									N/A
	Spouse	N/A		8 (88)					
	Adult child	N/A		1 (11)					
**Employment status of patients and informal caregivers, n (%)**									N/A
	Employed full time	2 (15)		3 (33)					
	Employed part time	2 (15)		1 (11)					
	Retired	7 (53)		2 (22)					
	Disability support	N/A		2 (22)					
	Sick leave	2 (15)		N/A					
	Missing	N/A		1 (11)					
**Health care profession, n (%)**			N/A		N/A				
	Nurse						7 (77)		
	Surgeon						2 (22)		
**Work experience in CRC treatment and care (years), n (%)**			N/A		N/A				
	1-3						5 (55)		
	4-7						2 (22)		
	>10						2 (22)		

^a^CRC: colorectal cancer.

^b^HCP: health care professionals.

^c^N/A: not applicable.

### Seeking Health Information

The first theme concerns *health information sources* and *using the internet to access health information*.

The patients and informal caregivers obtained information on illness and treatment from several different sources. One of the main sources was written information provided during hospitalization and at discharge. Both patients and informal caregivers preferred speaking to HCPs about their concerns and needs, especially the coordinator for the cancer treatment pathway, who followed up the patients throughout diagnosis and treatment:

They said if there is anything you wonder about, some questions, please call us! We have a contact person and a telephone number directly to the ward. Then we feel safe.

Informal caregiver, Interview 5

Some of the patients stated that turning to *a real person* for information was preferable for getting the message across and avoiding misunderstandings:

We must not replace the human factor with those smartphones. That makes me worried! I value a phone number much more than a URL... [Smiling] Gosh, now I feel old!

Patient, Interview 10

The patients were divided in their perceptions about using the internet to access health-related information about their cancer diagnosis and treatment. One of the patients said enthusiastically that it was her responsibility to keep herself informed and described how she used the internet to gain knowledge:

I think it is important. I google. Now I google a lot on cancer markers. I should know something about it, since it’s very new to me. I use the iPhone for everything. Read journals, read about the epicrisis, and retrieved information from them. I think it’s the right way to go, very important, so let’s talk about it!

Patient, Interview 2

Other patients were skeptical about searching for web-based information and about what they read on the internet. “Everyone is a google-doctor these days!” one patient said. Patients feared that the uncritical use of internet sources to access health information might lead to health anxiety. When asked if he used the internet to access health information, one patient answered:

No! For the simple reason that there is so much on that internet, you’ll get sick just from reading it. I try to relate to the information I get from the hospital and my GP, easy and simple! If you start reading...before you know it, you’ll have one foot in the grave. I’m sorry, but I’m against it.

Patient, Interview 10

Several patients and informal caregivers said that they searched for health information on a need-to-know basis, claiming that it was not in their interest to search for more information. Others became inclined to distance themselves from the cancer diagnosis as soon as they had their tumor surgically removed. In the HCP’s view, the patients’ need to search for web-based information was, in many cases, determined by diagnosis and the outcome of the surgical treatment:

After receiving information from us, the vast majority of our patients go home thinking that they are healed. It is a positive cancer group we work with. They are so super ready to get well! “Get it out of my body, I want it to be gone!” Then they hope it’s gone, and for many of them, the cancer is gone.

HCP, interview 2

The idea is that once you have removed a cancerous tumour, you should be able to be yourself again.

HCP, Interview 4

### Factors Affecting the Use of Information Technology

The second theme is made up of the following 3 categories: *The age dimension*, *Lack of digital competence*, and *Support to find relevant information*. The participants talked about several factors that might contribute to patients’ and informal caregivers’ actual use of eHealth solutions, and the age factor was mentioned by nearly all the participants. HCPs shared stories of how the patients, young and old, brought their smartphones and tablets to the hospital and used them to google symptoms or manage medical appointments. The majority of comments referred to old age as preventing patients and informal caregivers from using the technology. Although some pointed toward an emerging digital era within health care services, a generational change was thought necessary before one would see an increase in the use of eHealth:

We’re in a transition phase. Eventually, those who are older...they do not even know what Facebook is, but in 10 years’ time the situation will be different, everyone will have Facebook then, and will know how to use a computer.

HCP, Interview 3

This opinion was shared by patients who claimed that they were probably the last generation not to use ICT. Not all respondents thought of old age as an inhibiting factor for technology use, but they highlighted a lack of technology experience and low digital competence as possibly greater contributing factors:

To get hold of digital information is fine for me, but among my own age group, there has been a complaint that you do not get the pension on paper anymore. We are probably in a transition period where a generation is dying out in which some people have had jobs where technology has not been so prominent, and then we have the new generations to come. When our generation is gone, I think everything can go digital.

Patient, Interview 2

A second factor highlighted by participants was how lack of experience and interest in information technology may affect behavior and habits in the use of web-based health information. Some of the patients and informal caregivers expressed no interest in using the internet to access health information and were satisfied with more traditional information sources, such as written and oral information provided by the cancer pathway coordinator, the surgeon, or their general practitioner (GP). They also expressed uncertainty and concern about having to answer questions about cookies and how to get past them:

And it often pops up, like...ehh...“accept”, right? Then I do not always know what it is, so I do nothing. Is there something to accept? Does it matter, the cookie stuff?

Patient, Interview 1

Patients also shared stories of having been more or less forced into using ICT through work, by eager children or grandchildren, or by the digitalization of welfare services:

I had to. I have not been interested in it, but then I had to. Banks, bills, things like that. So, I felt I had to.

Patient, Interview 9

Some patients spoke of how the introduction of technology in the work context gave them valuable experience of information technology, which would help them become informed patients:

I was lucky and was part of a workplace where we got computers in the 80s. It has been the key to success. If you are involved in systems and are willing to change, then you will succeed.

Patient, Interview 6

None of the patients or informal caregivers had received advice from their HCP on the secure use of the internet to access health information, and only a few reported having been asked by their HCP whether they had accessed the internet for information related to the CRC and treatment before hospitalization. One of the informal caregivers said:

They [the HCP] probably thought he was too old, so it was never mentioned.

Informal caregiver, Interview 7

One patient was advised by an HCP not to search too much for web-based health information, whereas another expressed the need for guidance on secure web pages outside the patient information platforms:

It would have been very helpful, because you spend a lot of time searching for information you trust. Okay, you have the patient information platform where you can find the information that is about you, but otherwise, no!

Patient, Interview 6

HCPs found that many patients asked for information on how to log on to the internet. As a result, written login-information was included as part of the pretreatment information at admission. In general, the lack of guidance on accessing web-based health information was confirmed by the HCPs:

I think they (the patients) google a bit, but I have not asked them specifically if they have actually searched for information about the disease.

HCP, Interview 1

### Future eHealth Services for Patients With CRC

This theme comprises the categories *Content of health services*, *eHealth service quality*, *User interface (UI) of the service*, and *Delivering eHealth*. Thoughts on the content and functions of future eHealth services in CRC care were mainly expressed by patients and HCPs.

They explained how patients worry in the presurgical phase, and was suggested that comprehensive information on CRC and its treatments should be available not only to manage symptoms and bodily changes following hospital discharge but also to prepare for surgery:

I often use pictures to describe what we (surgeons) do. It could just as easily have been animated; I think. They could watch a 10-minute film clip...And something about follow-up, what is the usual follow-up with hospital checks, a little about wound treatment. There are probably many who wonder...when can I have a shower, (how to) keep the wound dry, how long should the staples or the stitches be left in? When to remove these strips, and stuff like that. When to contact a doctor? In terms of infection, what is common?

HCP, Interview 9

Presurgical worry was confirmed by several of the patients who said they had many questions and did not know what to expect:

I think it would be great if it [the eHealth application] contained everything the doctors explain, in different ways. What are the steps, what are the expectations, what can happen, how can you contribute yourself, what are the risks? If you get an infection, what then? Everything we’ve talked about could be in it.

Patient, Interview 6

Several patients and HCPs proposed that an eHealth service could contain lifestyle advice on matters such as diet and physical activity and how to deal with family matters, especially for patients with small children. Informal caregivers expressed the need for information on how to help the patient recover, be able to ask questions, and get an answer from an HCP:

Let’s say you could send a message or an email to the doctor, and you could get an answer, not necessarily the same day, but say in a few days, it would be absolutely fantastic!

Informal caregiver, Interview 3

One of the HCPs explained how a chat function using an avatar could be designed:

You could actually enter the chat, down there. Then a face of a person comes up...who you chat with, and then you can write your question, there and then.

HCP

Some participants suggested using chat functions as a way of getting emotional support and ventilating frustration and anxiety. One HCP explained how she often facilitated conversations between patients and support personnel, such as the hospital chaplain, and how digital chat function might provide support from professional informal caregivers following hospital discharge:

In the weeks following surgery, I think there is a lot of pondering among patients. So, somewhere they could to talk to another person and not clam up...Someone who can share their burden.

HCP, Interview 3

Regarding the management of illness and follow-up treatment at home, the participants proposed service functions that might ease the transition from in-patient to home. They suggested the use of checklists and patient cases or patient histories to monitor their health condition and obtain advice on how to deal with symptoms:

They could make [patient] cases. Then you could enter your own symptoms, like that and like that, and then it [the advice] would come up.

Patient, Interview 7

As a rule, they need a checklist. What should one really be aware of? The skin around the ostomy for example, or “How much have I had to drink today? Because now I’ve been admitted with dehydration again.” Or “What is really normal when it comes to ileostomy or colon ostomy output?” Yes, a checklist could have been helpful.

HCP, Interview 5

Both patients and HCPs suggested that reminders by SMS be included in the eHealth service to help with the administration of medical appointments. One patient said there was a need for a reminder function related to the 5-year follow-up plan, providing the time and place of the appointment, coordinated with the general practitioners’ appointments, required blood tests, and computerized tomography scanning. This idea was supported by HCPs:

I think there are many good things about it [an eHealth follow-up service], such as a text message notification about your medical appointments. I think it is very good. A lot of people feel stressed about it: “When is it?” “Where did I put the note?” “Were there any changes?” Then you get a reminder a day or two before the appointment. I think it seems very safe and good, so it’s nice stuff!

HCP, Interview 3

The participants were concerned with the quality and relevance of eHealth service functions. Information on cancer illness and treatments had to be easily perceived and updated to be acknowledged as relevant. In the HCP’s experience, patients read and perceived the information they received very literally; so, the information had to be relevant and up to date. Otherwise, the patients would perceive the information as incorrect or contradictory and would become frustrated and confused. Some of the HCPs experienced information provision as complex:

When we inform, it is a little generous maybe, with good intentions. It is difficult to get things detailed enough, and at the same time, sufficiently universal.

HCP, Interview 3

This was confirmed by one of the patients, who experienced difficulty in the fact that different treatments required different information:

Before the operation, there were many questions. Of course, that app could contain some facts. But again, some people have large parts of their intestine removed, others only a piece. How much should they [HCP] write? To write something that will capture everyone’s experience, you have to write in general terms. Otherwise, you have to write in detail about lots of different things, and people will be confused as to what applies to them and what does not apply to them.

Patient, Interview 5

The UI of an eHealth service raised some concerns among the participants as to whether the application should be accessed through smartphones, tablets, or computers or connected to an existing public eHealth platform. They highlighted the importance of considering how most people used digital devices, that the UI requirements should be adapted to the user’s digital competence and skills, and that not all patients would benefit equally from an eHealth service:

I think an app will be easiest for most people, considering that most people have a smartphone or a tablet.

Patient, interview 2

I imagine they have to have their own tablet. Or should there be something lying on each bedside table? Should there be apps for mobiles? Yes, most people have fancy phones, but then you have those who do not. Should you have a paper version for some people? I don’t know, actually, but it’s an interesting question. We live in a technological world.

HCP, Interview 2

In the final category, the HCP wondered whether an eHealth service provided by professionals in the specialist health care service would be too time consuming. In their view, including an eHealth service in a busy clinical practice might turn out to be too demanding to manage. They suggested that future eHealth services could benefit from having dedicated personnel to deliver the service.

## Discussion

### Principal Findings

The primary aim of this study is to explore the views of patients with CRC, their informal caregivers, and HCPs on ICT for CRC management and their thoughts on future eHealth services for supporting patients and informal caregivers through the CRC treatment pathway and follow-up. Overall, the participants of this study contributed to an increased understanding of digital information for health and highlighted the important aspects to be considered when designing eHealth services for patients treated for CRC and their informal caregivers. The first overarching theme demonstrated the web-based information-seeking behavior of patients and informal caregivers and how it may depend on individual characteristics (eg, age, digital competence), the ability or inclination to trust web-based health information, and whether HCPs facilitate the use of web-based resources to gain knowledge about the cancer diagnosis and its treatment. These conditions appear to be intertwined and must be seen in relation to each other, in the sense that lack of digital competence and guidance on how to use the internet to find health-related information can create uncertainty regarding the quality and relevance of the information, leading one to question whether one can trust the information and its source. Trustworthiness in seeking web-based health information was found to rely on the expertise of the website authors; the quality of information; and the patient’s age, sex, and perceived health status [30].

The second theme identified relevant content for a future eHealth service, not only to support patients’ self-management after surgery but also in the presurgical phase to ameliorate presurgical worry. The delivery of relevant health information through a patient’s eHealth service may provide the patient with the level of health literacy needed to prepare for treatment, engage in discussions with the HCP on treatment options, and conduct necessary self-management at home after surgery [31]. Thus, this study suggests that an eHealth service for patients with CRC might be introduced to the patient early in the treatment pathway, preferably before primary surgery. Adequate cancer care relies on available information, and HCPs are encouraged to provide their patients with access to web-based information sources as a complement to oral and written information [32]. To achieve this, the HCP needs to have the necessary skills and resources to access relevant web-based health information [33].

The participants suggested a range of features for an eHealth service that could meet the support needs of patients with CRC and informal caregivers. The desired features proposed by the participants included communicable elements, such as a chat function to meet the patient’s need for multifaceted informational support for their medical condition and emotional support to cope with the cancer diagnosis. Findings from the second theme also recommend the development of eHealth services with high acceptability and an appropriate UI. The current extensive use of smartphones and tablets among hospitalized patients was confirmed by the interviewees and provides the context for choosing interfaces for eHealth services. In 2017, approximately 342 million people were registered as mobile phone users in Western Europe [34]. This opens up a new scenario for eHealth designers. A design that focuses on user-friendliness, is intuitive, and provides accurate and easily accessible information will be required by future users of eHealth services [35]. This study was performed in a country with high internet access and use [15], a social good not available to everyone in a global context. In the *2030 Agenda for Sustainable Development*, the United Nations made “universal and affordable access to the Internet in the least developed countries by 2020” as one of their development goals [36]. Successful goal achievement may increase the uptake and use of smartphones and facilitate active interest in personal health care [37].

### Comparison With Prior Work

Finding, accessing, and understanding the required health information are among the self-management tasks that cancer patients associate with a negative impact on their daily life and well-being [38]. Age was described as a contributing factor in the use of the internet to find health information. Younger age can be a benefit for internet use [16], and a recent study of 9005 chronically ill individuals confirms that the use of digital information technologies to obtain health information declines with age [38]. Regarding patients with CRC, Wieldraaijer et al [13] found that younger patients (<65 years) searched for health information themselves more often than older patients who usually consulted their HCPs more. Although older users of ICT should be considered a heterogeneous group [39], HCPs are encouraged to provide both instrumental and social support to engage older cancer patients in accessing and using internet-based health information, such as individually tailored education and training, and facilitate the use of health technology [38].

The findings on the design of eHealth services to support patients with CRC highlight that technology acceptance and usefulness are important aspects to consider and that variations in user acceptance and engagement can be expected. Nadal et al [40] proposed a continuum of mobile health technology acceptance to be applied in the health domain, where the individual moves through pre- and postadoption phases of technology. The participants in this study reported high availability of mobile devices among patients with CRC. This finding suggests an increased familiarity with mobile phone use among patients with CRC to access digital health apps, which may be an advantage when introducing mobile health to patients, where one can expect most patients to have moved past the perceived ease-of-use phase of the technology acceptance cycle [40].

The second overarching theme reveals the content of remote eHealth services that the stakeholders find supportive and useful, not only following surgical treatment but also in the presurgical phase. Presurgical worry was reported by the majority of the participants as an issue to be targeted using eHealth. This finding supports earlier research findings that eHealth is useful not only for postsurgical follow-up but also throughout the CRC trajectory. Chapman et al [41] suggested that patients with CRC benefit from presurgical information and education delivered by smartphones and tablets, which are found to improve QoL and mental health.

Furthermore, this study shows that patients and informal caregivers are in need of contact with health care specialists following discharge for the patient to recover well and to engage in the recovery process by self-monitoring and taking action for health improvement. Our findings suggest that future CRC eHealth services may be offered to patients at discharge to facilitate communication with HCPs in the early stages of recovery. Drott et al [9] showed that patients with CRC experienced increased engagement in self-management by using smartphones to communicate treatment side effects to clinicians. On the other hand, this study reveals that from the HCP’s point of view, a follow-up eHealth service offered for CRC at discharge would be too time consuming. As a result of the social distancing required by the COVID-19 pandemic, eHealth solutions (eg, video consultations) have been increasingly used in specialist health care services [42] and may represent a changed view of the use of technology in health care delivery. In designing future eHealth solutions, it is important to consider both patients and HCP as users of the technology and involve them in discussions regarding the area of use and service delivery.

The strengths of this study include a multiple-perspective approach to data collection and the use of a validated data analysis framework [28]. A range of eHealth application features were proposed by the participants, which provide valuable input into the design of future eHealth services. Targeting the needs of end users before introducing them to the technology is crucial for ensuring high levels of usability and user satisfaction [24]. The use of a purposive sampling technique secured participant samples rich in information on CRC treatment and care and living with CRC [43]. A continuous evaluation of sample size adequacy was applied during data collection, following Malterud et al [26] model of information power.

### Limitations

This study has some limitations. This study involved only one study site. Recruiting from multiple study sites might have resulted in a more varied participant sample, ensuring the generalizability and external validity of the findings [44]. The majority of HCPs were women and nurses. Future research may benefit from a more balanced HCP sample with regard to sex and work profession. For practical reasons, most informal caregivers were interviewed by telephone, which may have yielded less rich data from this sample. Although telephonic interviews may create a bias resulting in loss of data and lower quality of findings, they may also save resources and provide access to geographically disparate participants, as shown in this study [45]. The study was performed in a context dominated by high internet access and use; thus, the study findings may not be generalizable to regions with low internet access.

### Conclusions

This study shows that the increasing use of the internet to manage serious illnesses and treatments, digitalization of health care services, and engagement of stakeholders (ie, patients with CRC, informal caregivers, and HCP). Several elements come into play and affect the health-information-seeking behavior of patients with CRC and their informal caregivers. Age and lower digital competence may hinder the patients from accessing web-based health information. HCPs report a shift in the approach of patients with CRC to gaining health information through web-based channels, but patients are seldom guided toward accessing web-based health information that is trustworthy and of high quality. An eHealth service for patients with CRC may comprise elements of information, education, and support for self-management of pre- and postsurgical treatment and should be adapted to the patient’s age and digital competence. Cocreation of eHealth services with stakeholders is recommended to ensure tailored services of high quality that are perceived as user friendly and valuable by end users.

## Appendix

Multimedia Appendix 1 Display of data analysis according to systematic text condensation steps 1 to 4.

## Abbreviations

CRC: colorectal cancer
HCP: health care professional
ICT: information and communication technology
QoL: quality of life
UI: user interface
