# Supplementary material for: Stakeholders’ Perspectives on eHealth Support in Colorectal Cancer Survivorship: Qualitative Interview Study
Source: JMIR Cancer. 2021 Sep 7;7(3):e28279. doi: 10.2196/28279 (PMC8456333; doi:10.2196/28279)
Supplement: Multimedia Appendix 1 [file cancer_v7i3e28279_app1.docx]

MULTIMEDIA APPENDIX 1

**Table 2** Display of data analysis according to Systematic Text Condensation step 1-4

| **STEP 1 Pre-liminary themes** | **STEP 2  Meaning Units (an example)** | **STEP 3 Sub-categories** | **STEP 4 Main categories** |
| --- | --- | --- | --- |
| Obtaining health-related information    Purpose of using information technology | There is so much on the internet, you get sick just by reading it all. I try to relate to the information I get from the hospital and my GP, easy and simple. There are many “google doctors”, if you start reading you suddenly stand with one foot in the grave. I'm sorry, but I'm against it (Patient).  I google, and right now I google a lot on cancer markers (Patient) | Health information sources  Using internet to access health information | Seeking health information on digital platforms |
| Prerequisites for using information technology  eHealth services design and usefulness | I'm too old and not been interested. I was old when I had to learn it (using health technology). It is something else for those who grow up with it, they know everything (Patient)  I've got it [a tablet] for Christmas. You know, they [the children] do not know what to give me for Christmas. I'm playing in an orchestra, they said I had to go on Facebook to get information. But it does not work. I don’t use it much (Patient)  A lot of people use it (the internet). We get daily questions: How do I log on to the internet? That is why we have made an instruction to hand out to them, because we know they will ask for it (HCP) | The age dimensions  Lack of digital competence  Support to find relevant information’ | Factors affecting the use of information technology |
|  | When it comes to the wounds and such, or if I am in pain, I could have chatted with someone in the hospital, who could look at my medical history, who has been through this many times before, then they could answer this (Patient)  If it was sites that could help them, then I think it would have been good, but if you google any possible symptom, then you can be a little scared, you will find quite a lot about diseases and such. But if it used help pages, for example the Cancer Society’s site and things like that, then I think it would have been helpful (HCP)  It is important to make things simple, it must not be too complicated (Carer)  I wonder, who will sit and answer the messages, because in a very busy ward we spend a lot of time on the patients when they are admitted, and answering a huge amount of calls from relatives… It's pretty much all the time (HCP) | Content of health services’  eHealth service quality’  User interface of the service  Delivering eHealth | Future eHealth services for colorectal cancer patients |
